# El Tratamiento con Bloqueadores del Sistema Renina-Angiotensina-Aldosterona no debe suspenderse tras la Infección por SARS-CoV-2

**DOI:** 10.47487/apcyccv.v1i2.62

**Published:** 2020-06-29

**Authors:** Alfonso Bryce-Moncloa, Arianna Portmann-Baracco, Mayte Bryce-Alberti

**Affiliations:** 1 Unidad de Investigación CARDIOGOLF - Lima, Perú Unidad de Investigación CARDIOGOLF Lima Perú; 2 Expresidente de la Sociedad Peruana de Hipertensión Arterial Expresidente de la Sociedad Peruana de Hipertensión Arterial; 3 Universidad Peruana Cayetano Heredia. Lima, Perú Universidad Peruana Cayetano Heredia Universidad Peruana Cayetano Heredia Lima Peru

## Introducción

En el transcurso de tres meses, la pandemia COVID-19, ha llegado a millones de personas a nivel mundial y continúa superando las capacidades de los sistemas de salud. Con la promesa de una vacuna que podría estar disponible recién en 12 a 18 meses, el enfoque principal de las autoridades ha sido a nivel de medidas de prevención para evitar el contagio por SARS-CoV-2. ^(^^1^^)^ No obstante, se han identificado factores de riesgo para contraer la enfermedad y para desarrollar estadios severos de esta. Evidencia científica identifica la hipertensión arterial (HTA) como la comorbilidad más frecuente en pacientes COVID-19, particularmente en aquellos de edad avanzada. ^(^^2^^,^^3^^)^ Asimismo, este grupo de pacientes tiende a progresar a la fase severa de la enfermedad, caracterizada por hiperinflamación, alteraciones en la cascada de coagulación y daño multiorgánico. ^(^^4^^,^^5^^)^ Dado que la enzima convertidora de angiotensina 2 (ECA2) es el receptor que permite la entrada del SARS-CoV-2 a la célula huésped, ^(^^6^^)^ surgió la preocupación de que el uso preexistente de bloqueadores del sistema renina-angiotensina-aldosterona (SRAA), podría aumentar el riesgo de desarrollar una infección grave y mortal por este virus.

## La comorbilidad más frecuente en COVID-19 

Como se comentó previamente, se ha reportado a la HTA como la comorbilidad más frecuente en pacientes COVID-19. Singh *et al.* ^(^^2^^)^ y Schiffrin *et al*, ^(^^3^^)^ reportaron una prevalencia de esta, de 21% y 30%, respectivamente; sin embargo, aún no es claro si la HTA, al igual que otras comorbilidades frecuentes como la diabetes mellitus y la enfermedad coronaria, son solo hallazgos epidemiológicos entre una población de adultos mayores; alternativamente, la edad en sí, podría ser el factor de riesgo más importante para COVID-19. ^(^^3^^)^

Si bien, no se ha establecido con claridad una relación causal entre HTA e infección por SARS-CoV-2, o si la presión alta es un factor de riesgo para COVID-19, ya se conoce que el control de la presión arterial es importante en el manejo del paciente infectado. ^(^^7^^)^ Asimismo, se ha reportado mayor riesgo de desarrollar COVID-19 severo y mayor mortalidad en pacientes hipertensos en comparación con no hipertensos. ^(^^4^

## Fisiopatología del COVID-19 y el SRAA

Para entender la fisiopatología de la enfermedad desde su ingreso a la célula, replicación viral, hasta la fase más severa caracterizada por una tormenta de citoquinas, es importante entender el rol que cumple el SRAA en esta infección. Esta vía comienza con la producción del angiotensinógeno en el hígado y su conversión a angiotensina (Ang) I por acción de la Renina. La enzima convertidora de angiotensina (ECA) convierte la Ang I en Ang II y esta última es convertida a Ang 1-7 por acción de la ECA2, la cual además sirve como receptor del SARS-CoV-2. Es importante diferenciar la ECA de la ECA2, ya que ambas ejercen acciones muy diferentes que llevan a mecanismo contrarios. Mientras que la Ang II, producida por la ECA, es un mediador proinflamatorio, pro-fibrótico y vasoconstrictor; ^(^^8^^)^ la Ang 1-7, producida por la ECA2 libre, se une al receptor de la vía MAS^9^^)^ y produce efectos vasodilatadores, antiinflamatorios, antioxidantes, antifibróticos^10^^)^ y cardioprotectores. ^(^^11^

La ECA y la ECA2 producen un balance del SRAA entre los mecanismos inflamatorios y antiinflamatorios. El virus SARS-CoV-2, al unirse al receptor ECA2 en el tejido pulmonar, ingresa a la célula a través de internalización del complejo formado por el virus y el receptor. Esto produce una disminución del ECA2 celular y, por lo tanto, de ECA2 libre. En consecuencia, se produce una disregulación del SRAA hacia la vía inflamatoria por el exceso Ang II, lo que produce una severa vasoconstricción, aumento de la permeabilidad vascular, inflamación severa y fibrosis intersticial que explicaría la gravedad del daño que se produce en el pulmón. ^(^^10^^)^ Estos mecanismos han llevado a los científicos a discutir si el aumento del ECA2, observado en algunas comorbilidades y con el uso de inhibidores del SRAA, es perjudicial al aumentar la unión del virus al tejido pulmonar o si es beneficioso al disminuir el efecto inflamatorio y la propagación de la tormenta de citoquinas. 

## Antihipertensivos que bloquean el SRAA

Uno de los pilares del tratamiento de la HTA, son los inhibidores del SRAA: inhibidores de la enzima convertidora de angiotensina (IECA) y antagonistas del receptor de angiotensina II (ARA II). Estos medicamentos actúan disminuyendo o inhibiendo a la Ang II y, por lo tanto, sus efectos proinflamatorios. Parte de la confusión del público en general se debe a que no diferencian los inhibidores de la ECA con los inhibidores de la ECA2, siendo estas dos enzimas diferentes con sitios activos específicos para cada una. Se ha propuesto que estos medicamentos tienen un efecto protector al actuar de manera indirecta en el aumento de la expresión de los receptores de ECA2 y con ello, sus propiedades antiinflamatorias, antioxidativas y cardioprotectoras. ^(^^10^^)^

Adicionalmente, existen reportes limitados en animales que demuestran que los inhibidores del SRAA, sobre todo los ARA II, afectan la expresión de ECA2 en tejido cardíaco y vasculatura renal. El efecto sobre la ECA2, por parte de los ARA II, se ha documentado consistentemente en varios estudios a nivel de ARN mensajero y proteínas. No obstante, las dosis requeridas para generar este efecto son altas y la data es insuficiente para poder extrapolar los hallazgos a humanos. Por lo tanto, no se puede afirmar que estos medicamentos facilitan la entrada de SARS-CoV-2 a la célula huésped. ^(^^12^

Por el contrario, se destaca el efecto pulmonar potencialmente beneficioso de los ARA II. Durante la lesión pulmonar aguda, la ECA2 alveolar suele estar regulada negativamente por lo que el metabolismo de la Ang II también disminuye. Esto conlleva a mayores niveles locales del péptido y por ende, mayor permeabilidad alveolar y consecuente lesión pulmonar. En este contexto, al aumentar la expresión de ECA2 y por tanto, de Ang 1-7, mediante el tratamiento con ARA II preexistente, se estaría instaurando un efecto protector durante el curso de la infección por SARS-CoV-2. ^(^^10^^,^^12^

Los inhibidores del SRAA no solo satisfacen las recomendaciones de manejar la presión arterial en los pacientes COVID-19, sino que además, mitigan la tormenta inflamatoria propia de los estadios severos de la infección. Entonces, ante la duda de si estos beneficios superan el supuesto riesgo de aumento de ingreso de SARS-CoV-2 a las células, se han realizado estudios que demuestran que los pacientes con COVID-19 en tratamiento con inhibidores del SRAA no tienden a desarrollar enfermedad severa^13^^)^ y, por el contrario, protegen al paciente de injuria pulmonar a través de la conversión de Ang II a Ang 1-7. ^(^^10^

Con base a la producción científica hasta la fecha, no hay evidencia que soporte discontinuar el tratamiento antihipertensivo con IECA o ARA II. ^(^^14^^)^ Por esta razón, las sociedades expertas en el uso de antihipertensivos a nivel internacional como International Society of Hypertension, ^(^^15^^)^ European Society of Cardiology, ^(^^16^^)^ American Heart Association, American College of Cardiology, Heart Failure Society of America, ^(^^17^^)^ Canadian Cardiovascular Society, ^(^^18^^)^ High Blood Pressure Research Council of Australia, ^(^^19^^)^ entre otras, apoyan el uso de IECA y ARA II en los pacientes hipertensos con COVID-19 y han tomado una posición en contra de su discontinuación durante la enfermedad.

## Conclusión

La HTA es la comorbilidad más frecuente en los pacientes COVID-19. En ellos, se ha visto un aumento de progresión a formas de infección por SARS-CoV-2 severa y mayor mortalidad. Adicionalmente, en la actualidad, no se ha encontrado ninguna razón para abandonar o suspender temporalmente el uso de los inhibidores del SRAA en los pacientes hipertensos, como forma preventiva en pacientes COVID-19. Estos antihipertensivos son de importancia crítica y su beneficio terapéutico supera cualquier riesgo potencial de predisposición a la infección por SARS-CoV-2.
